# Polymorphic Variants of SCN1A and EPHX1 Influence Plasma Carbamazepine Concentration, Metabolism and Pharmacoresistance in a Population of Kosovar Albanian Epileptic Patients

**DOI:** 10.1371/journal.pone.0142408

**Published:** 2015-11-10

**Authors:** Armond Daci, Giangiacomo Beretta, Driton Vllasaliu, Aida Shala, Valbona Govori, Giuseppe Danilo Norata, Shaip Krasniqi

**Affiliations:** 1 Department of Pharmacy, Faculty of Medicine, University of Prishtina, Prishtina, Kosovo; 2 Institute of Pharmacology and Toxicology and Clinical Pharmacology, Faculty of Medicine, University of Prishtina, Prishtina, Kosovo; 3 Department of Pharmaceutical Sciences, Università degli Studi di Milano, Milan, Italy; 4 University of Lincoln, School of Pharmacy, Joseph Banks Laboratories, Green Lane, Lincoln, LN6 7DL, United Kingdom; 5 Neurology Clinic, University Clinical Center of Kosova, Prishtina, Kosovo; 6 Department of Pharmacological and Biomolecular Sciences, Università degli Studi di Milano, Milan, Italy; 7 Center for the Study of Atherosclerosis, Ospedale Bassini, Cinisello Balsamo, Italy; Geisel School of Medicine at Dartmouth College, UNITED STATES

## Abstract

**Aim:**

The present study aimed to evaluate the effects of gene variants in key genes influencing pharmacokinetic and pharmacodynamic of carbamazepine (CBZ) on the response in patients with epilepsy.

**Materials & Methods:**

Five SNPs in two candidate genes influencing CBZ transport and metabolism, namely ABCB1 or EPHX1, and CBZ response SCN1A (sodium channel) were genotyped in 145 epileptic patients treated with CBZ as monotherapy and 100 age and sex matched healthy controls. Plasma concentrations of CBZ, carbamazepine-10,11-epoxide (CBZE) and carbamazepine-10,11-trans dihydrodiol (CBZD) were determined by HPLC-UV-DAD and adjusted for CBZ dosage/kg of body weight.

**Results:**

The presence of the SCN1A IVS5-91G>A variant allele is associated with increased epilepsy susceptibility. Furthermore, carriers of the SCN1A IVS5-91G>A variant or of EPHX1 c.337T>C variant presented significantly lower levels of plasma CBZ compared to carriers of the common alleles (0.71±0.28 vs 1.11±0.69 μg/mL per mg/Kg for SCN1A IVS5-91 AA vs GG and 0.76±0.16 vs 0.94±0.49 μg/mL per mg/Kg for EPHX1 c.337 CC vs TT; P<0.05 for both). Carriers of the EPHX1 c.416A>G showed a reduced microsomal epoxide hydrolase activity as reflected by a significantly decreased ratio of CBZD to CBZ (0.13±0.08 to 0.26±0.17, p<0.05) also of CBZD to CBZE (1.74±1.06 to 3.08±2.90; P<0.05) and CDR_CBZD_ (0.13±0.08 vs 0.24±0.19 μg/mL per mg/Kg; P<0.05). ABCB1 3455C>T SNP and SCN1A 3148A>G variants were not associated with significant changes in CBZ pharmacokinetic. Patients resistant to CBZ treatment showed increased dosage of CBZ (657±285 vs 489±231 mg/day; P<0.001) but also increased plasma levels of CBZ (9.84±4.37 vs 7.41±3.43 μg/mL; P<0.001) compared to patients responsive to CBZ treatment. CBZ resistance was not related to any of the SNPs investigated.

**Conclusions:**

The SCN1A IVS5-91G>A SNP is associated with susceptibility to epilepsy. SNPs in EPHX1 gene are influencing CBZ metabolism and disposition. CBZ plasma levels are not an indicator of resistance to the therapy.

## Introduction

Epilepsy is a disease that cannot be described by only a single condition, but it rather represents a family of diverse disorders, having in common an abnormally increased predisposition to seizures, which occur due to abnormal, excessive or synchronous brain neuronal activity [1]. Prevalence of epilepsy is higher in developing countries and also slightly higher in lower socioeconomic classes. It occurs in all strata in a population, and males are more predisposed compared to females. About 40% of patients develop epilepsy below the age of 16 years and about 20% over the age of 65 years, with a frequency that different studies have shown to vary between 50 and 120 per 100,000 individuals per year [2]. Carbamazepine (CBZ) belongs to one of the most prescribed anticonvulsant drugs for treatment of generalized tonico-clonic and complex partial epileptic seizures [3]. As several other antiepileptic drugs, CBZ is a substrate of the human P-glycoprotein (Pgp) transporter [4]. CBZ is metabolized in the liver through an oxidative, epoxidase pathway catalyzed by CYP3A4 and other CYP enzymes followed by epoxide hydrolase mediated pathway. This leads to the formation of CBZ-10,11-epoxide (CBZ-E), the major CBZ metabolite, which possesses a potent anticonvulsant effect, before further metabolism by microsomal epoxide hydrolase (mEH) and excretion as inactive CBZ-10,11-diol (CBZ-diol) [5,6].

From the pharmacological point of view, CBZ exerts a combined antiepileptic action by use-dependent blockage of neuronal sodium channels in a voltage and frequency dependent manner by delaying their recovery from the inactivated state, through reduction of the number of action potentials within a burst and decrease of burst duration [7,8].

Clinically, CBZ is characterized by important inter and/or intraindividual variation in drug pharmacokinetics and by different patient susceptibility to adverse reactions. As a consequence, the therapeutic efficacy of CBZ, as well as those of other similar antiepileptic drugs, seems to correlate better with blood levels than with doses [9,10]. For these reasons, therapeutic drug monitoring emerged as an essential tool for therapy optimization and for minimizing the side effects arising due to excessive drug blood concentrations (or to avoid lack of pharmacological effect due to its lower than expected blood levels) [11].

In addition, and above all, as seizures can effectively be pharmacologically suppressed, a high percentage of patients (30–40%) exhibit pharmacoresistance independently from medication non-compliance, significant provoking factors, inappropriate drug or doses, or progressive neurological diseases [12,13]. The main proposed cause for CBZ pharmacoresistance is the genetic polymorphism existing in genes encoding for proteins associated with CBZ metabolizing enzymes (mediated by CYP3A4, CYP3A5, CYP2C9, CYP2C19 and EPHX1), transporter proteins (ABCB1, ABCC1), or target proteins and receptors (SCN1A, SCN2A). Understanding of these may enable prediction of drug resistance and optimization of therapeutic strategies [14–18]. However, studies investigating the effect of SNPs of a variety of genes on CBZ metabolism in different populations have achieved, in several cases, contradictory conclusions, probably due to geographical/genetically differences existing among the studied populations [19–28].

The present study aimed to evaluate the potential associations between SNPs of key genes encoding for the major drug transporter protein ABCB1, for the metabolizing enzyme EPHX1, and for the sodium channel SCN1A, as genes involved in the metabolism and disposition of CBZ, and CBZ plasma levels in epileptic patients treatment.

## Materials and Methods

### Subjects

All the procedures in this study were conducted according to guidelines in the Declaration of Helsinki and the study design was approved by Ethics Committee in Faculty of Medicine, University of Prishtina—Hasan Prishtina and University Clinical Center of Kosovo (Prishtina, Kosovo). All patients gave written informed consent. A total of 145 patients with epilepsy (82 males and 63 females) between the ages of 18–70 years were included in the study. Patients were treated with CBZ monotherapy for at least 1 year at Neurology Clinic in the University Clinical Center of Prishtina. All patients were not receiving pharmacological treatment for other pathologies. Patients’ renal and hepatic functions were evaluated and those with abnormal function were not included in the study. The classification of epilepsies and epileptic syndromes were conducted according to the guidelines of the International League Against Epilepsy [29]. A total of 100 unrelated healthy control individuals were also randomly recruited from the same region and ethnicity to compare genotyping distribution. All patient were from Kosovo and the following information was noted: gender, weight (kg), age, CBZ maintenance dose (mg/kg per day), drug resistant patients (considered as occurrence of at least four seizures over a period of 1 year during treatment with CBZ) and drug responsive patients (those seizure-free for at least 1 year during treatment with CBZ) (Table 1). No dose adjustments were allowed within 1 month prior to the collection of samples to ensure steady-state plasma concentrations of CBZ.

**Table 1:** Patient characteristics and dose-adjusted concentrations and reciprocal ratios of CBZ and its major metabolites CBZE and CBZD. CBZE: carbamazepine-10,11-epoxide; CBZD: 10,11-dihydroxy-carbazepine.

Characteristics	Patients	Controls
Total patients (n)	145	100
Sex (male/female)	82/63	58/42
Age (years)	32.9±15.5	30.4±14.4
Weight (Kg)	68.3±16.0	66.5±15.5
CBZ maintenance dose (mg/Kg per day)	8.18±3.91	-
Drug resistance/response	48/97	-
CBZ plasma concentration (μg/mL)	6.78±2.78	-
CDR_CBZ_ (μg/mL per mg/Kg)	0.56±0.55	-
CBZE plasma concentration	0.74±0.48	-
CDR_CBZE_ (μg/mL per mg/Kg)	0.24±0.18	-
CBZD plasma concentration	1.81±1.38	-
CDR_CBZD_ (μg/mL per mg/Kg)	0.24±0.18	-
CBZD:CBZE	2.99±2.57	-

A total of 6 mL of fasting peripheral blood was drawn early in the morning from each patient in EDTA and heparinized vacutainer blood collection tubes for DNA extraction and analysis of plasma concentrations of CBZ and of its major metabolites.

The maintenance dose-adjusted concentrations of CBZ, CBZE and CBZD (CDR_CBZ_, CDR_CBZE_, CDR_CBZD_) and the CBZE:CBZ, CBZD:CBZ and CBZD:CBZE ratios were used as parameters for the evaluation of CBZ metabolism.

### Genotyping

Genomic DNA was extracted from whole blood using a Purelink Genomic DNA extraction kit according to the procedure recommended by the manufacturer (Invitrogen, CA, USA). The genotypes of *ABCB1* c.3435C>T (rs1045642), SCN1A c.3184A>G (rs2298771), IVS5–91 G>A (rs3812718), and *EPHX1* c.416A>G (rs2234922), c.337T>C (rs1051740), polymorphisms were analyzed using SNP specific Taqman probes Vic and Fam reporter dyes, according to the manufacturer’s instructions (Applied Biosystems, Foster City, CA). DNA samples were diluted to a concentration of 10 ng/μL. The assays were run using a reaction volume of 15 μL, consisting of 7.5 μL of Applied Biosystems TaqMan Genotyping Master Mix, 0.75 μL of TaqMan SNP Genotyping Assays and Drug Metabolism Genotyping Assays, 1.75 μL of DNAse/RNAse free water and 5 μL of diluted DNA. Initial denaturation step was 7 minutes and 30 seconds, followed by 45 cycles of 15 at 92°C and anneal/extend for 1 min at 60°C on a iCycler iQ^™^ Real-Time PCR Detection System BioRad Machine.

### Determination of CBZ, CBZE and CBZD by HPLC-UV-DAD

Plasma samples for analyses were obtained from whole blood by centrifugation at 4000 rpm for 4 minutes. Measurement of plasma CBZ, CBZE and CBZD was carried out using an Ultra Fast Liquid Chromatographic System (Schimadzu-Japan).

Chromatographic separations were done using a reversed-phase column (KINETEX C18 5 μm, 150 x 4.6 mm i.d., Phenomenex, Castel Maggiore, Bologna, Italy), run in isocratic conditions with acetonitrile/water (20:80) mobile phase at a flow rate of 1.5 ml/min. Column temperature was 25°C. The DAD detector operated between 200 nm and 400 nm, and the monitoring wavelengths were set at λ = 285 nm for CBZ monitoring, λ = 250nm for phenacetin and λ = 215 nm for CBZE and CBZD. Method validation was developed following recommendation for validation of bioanalytical methods of European Medicine Agency guideline.

Sample preparation was carried out using solid-phase extraction. Calibration curves were built using blank plasma spiked with previously prepared standards for analyses from stock solutions of CBZ, CBZE and CBZD (Sigma-Aldrich). OASIS Hydrophylic-Lipophilic-Balanced sorbent cartridges (HLB, 30mg, Waters Corporation, Millford, MA) were used for extraction. Cartridges were first preconditioned with 1 mL of pure methanol, followed by washing out of the solvent with 1 mL of MilliQ water. 50 μL of IS solution (25 μg/mL phenacetin in methanol) was added to each 250 μL sample, followed by sample vortex-mixing for 30 seconds, centrifugation at 6000 G and supernatant loading on solid phase extraction cartridge. After washing with 1 mL of 5% methanol in MilliQ water, analytes were recovered with 500 μL of absolute methanol and 10 μL injected in the HPLC system.

### Statistical analysis

All data were expressed as mean and standard deviation (SD). Before statistical analysis, normal distribution and homogeneity of the variances were tested. Associations between the experimental parameters were investigated using one-way ANOVA, followed by *t*-tests on pairwise comparisons with the least square difference (LSD) *post hoc* adjustment for multiple comparisons. Apparent CBZ clearance (CL) was calculated according to the formula:

$$CL\;=\;F\frac{CBZ_{maintenance\;dose}}{CBZ_{plasma\;concentration}\;\times \left(\tau \right)}$$

where CBZ_plasma concentration_ is plasma CBZ concentration at steady state, F is bioavailability and (τ) is the dosing interval [30,31]. Genotype frequencies were checked with Hardy—Weinberg equilibrium using χ2 test. The relationship between various genotypes and responsiveness was examined using binary logistic regression. Associations were expressed as odds ratios (OR) or risk estimates with 95% confidence intervals (CI) and considered significant when *P*-value was <0.05. Statistical analysis was performed using the R-commander GUI for R (v. 3.1.3) [32].

## Results

### Quantitative determination of CBZ, metabolites and related parameters

**Fig 1:**
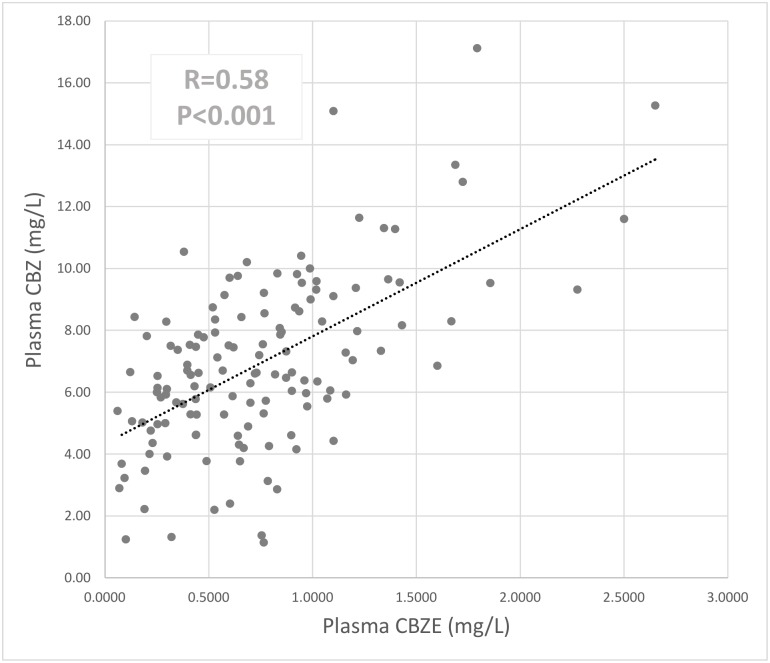
Correlation between CBZ and CBZE plasma concentrations (Pearson’s correlation coefficient R).

CBZ daily dose showed direct correlations with the plasma levels of both CBZ (R = 0.58, P<0.001) and CBZE (R = 0.37, P<0.001) (S1 Fig). Plasma concentrations of the active metabolite, CBZ-10,11-epoxide (CBZE), were significantly correlated to CBZ plasma levels (R = 0.58, P<0.001; Fig 1).

### Impact of sodium channel SNPs on the prevalence of epilepsy

**Fig 2:**
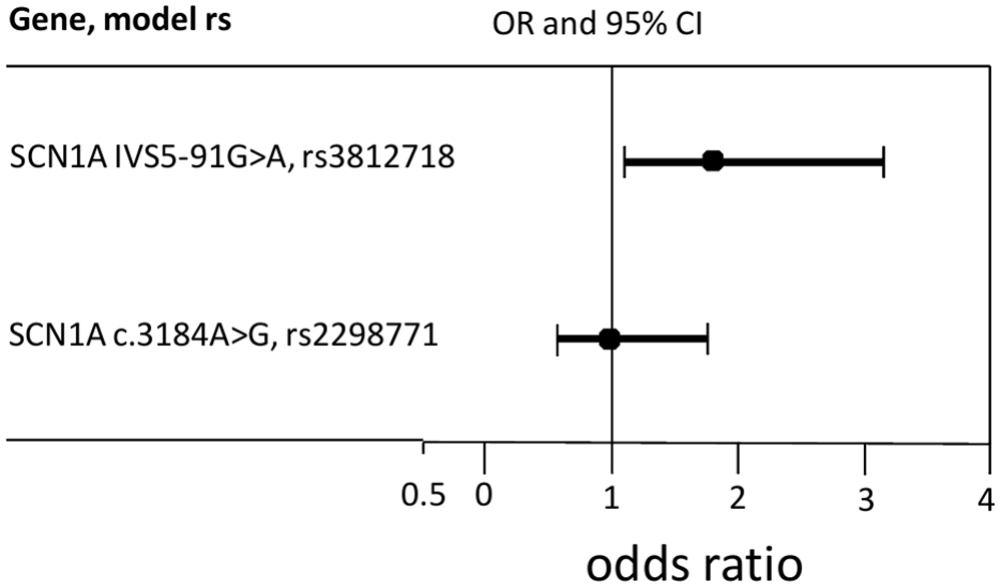
Graphical representation of adjusted odds ratio and 95% confidence intervals according to the SCN1A genes polymorphism; genotype and allele frequencies in epilepsy patients vs. healthy control subjects.

We first studied whether two SNPs in sodium channel (SNC1A) affect the prevalence of epilepsy. Genotyping these two SNPs in epileptic patients and healthy controls revealed that carriers of the SCN1A IVS5-91G>A variant were at increased risk of epilepsy susceptibility (P = 0.033; OR 1.80, 95% CI 1.048, 3.094), while no impact for SCN1A c.3184A>G SNP was observed (Fig 2). Data regarding the prevalence of all the SNPs investigated in epileptic patients compared to controls are shown in (S1 Table).

### Impact of gene variants in genes influencing CBZ metabolism and disposition parameters and CBZ metabolite plasma levels

**Fig 3:**
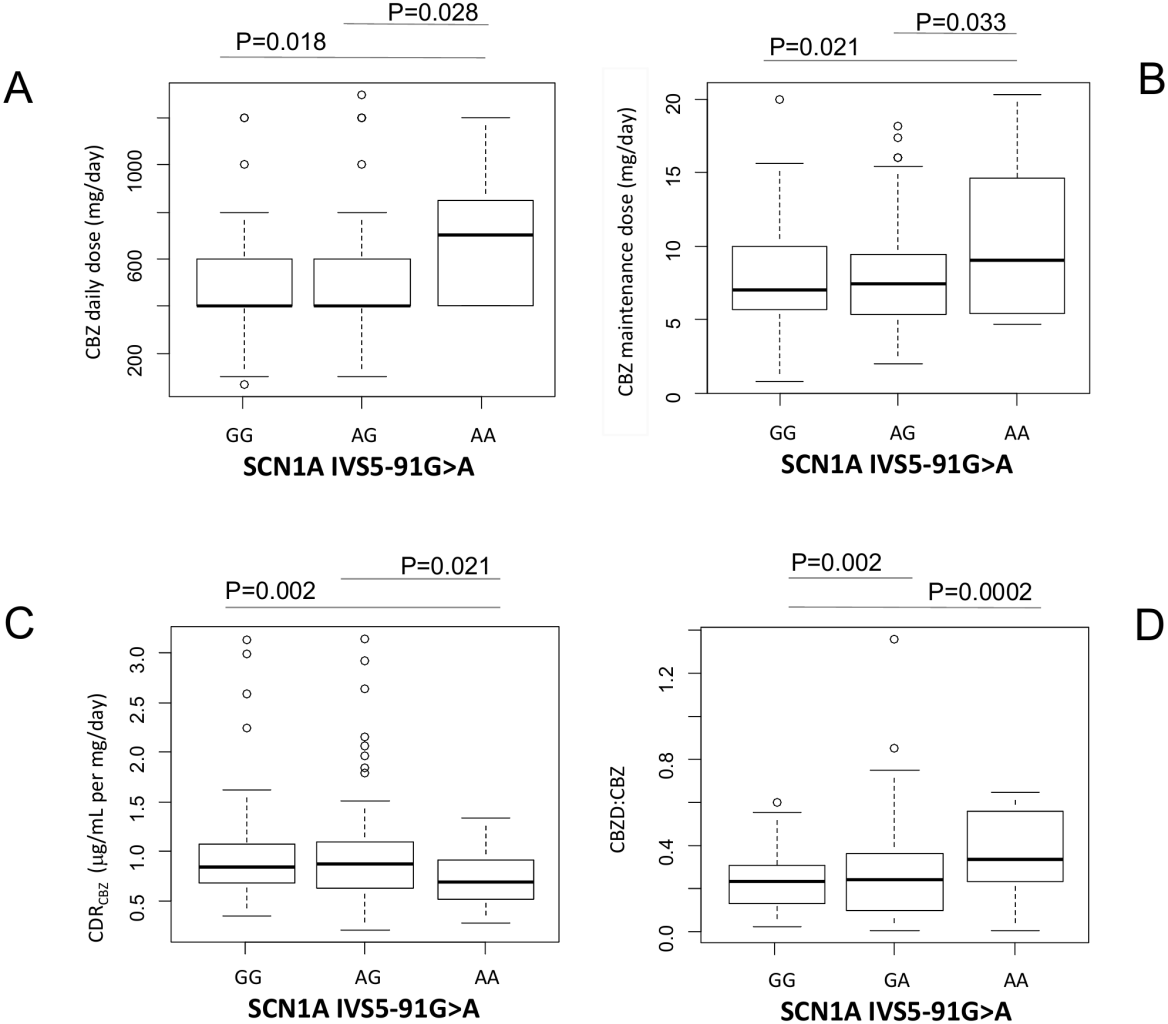
Graphical representations (box-plot) of the sodium channels (SCN1A IVS5-91 G>A) SNPs genotypes associations with (A) CBZ daily dosage (mg/day), (B) CBZ maintenance dose (mg/kg per day), (C) CDR_CBZ_ and (D) CBZD:CBZ. Statistical significance for difference of means is shown (P values, one way ANOVA analysis followed by Student’s T-test).

Patients carrying the AA variant/genotype of the SCN1A IVS5-91G>A gene showed increased maintenance dosage (Fig 3B), reduced CBZ plasma levels (Fig 3C) and increased CBZD to CBZ ratio (Fig 3D) (Table 2), despite taking higher CBZ daily dosage compared to GA or GG carriers (694±313 mg/day for AA compared to 509±248 mg/day for GA and 531±254 mg/day for GG. P<0.05, Fig 3A).

**Table 2:** CBZ daily dose, maintenance dose, concentration/dose adjusted ratios of CBZ, CBZE, CBZD and their concentration ratios stratified by individual SNPs genotypes. Data are mean ± standard deviations.

SNP	n	CBZ daily dose (mg/day)	CBZ maintenance dose (mg/Kg per day)	CDR_CBZ_ (μg/mL per mg/Kg)	CDR_CBZE_ (μg/mL per mg/Kg)	CDR_CBZD_ (μg/mL per mg/Kg)	CBZE:CBZ	CBZD:CBZ	CBZD:CBZE
**SCN1A IVS5-91G>A**									
**rs3812718**									
GG	50	509.3±248.0^a^	7.79±3.90^a^	1.11±0.69^a^	0.12±0.13^a^	0.23±0.15^a^	0.10±0.06^a^	0.23±0.13^a^	3.00±2.45^a^
GA	79	531.6±249.2^a^	7.96±3.46^a^	0.92±0.46^a^	0.10±0.07^a^	0.23±0.19^a^	0.13±0.12^a^	0.27±0.22^a^	2.78±2.44^a^
AA	16	693.8±313.0^b^ *	10.48±5.32^b^ *	0.71±0.28^b^ *	0.08±0.04^a^	0.28±0.10^a^	0.14±0.08^a^	0.43±0.17^b^ **	4.20±3.17^a^
**SCN1A c.3184A>G**									
**rs2298771**									
AA	25	524.0±238.5^a^	7.57±3.85^a^	1.13±0.71^a^	0.11±0.11^a^	0.22±0.14^a^	0.11±0.06^a^	0.22±0.14^a^	2.87±2.56^a^
AG	83	522.9±245.5^a^	8.06±3.66^a^	0.93±0.49^a^	0.10±0.08^a^	0.24±0.20^a^	0.12±0.12^a^	0.26±0.21^a^	2.98±2.59^a^
GG	37	596.4±313.8^a^	8.86±4.16^a^	0.93±0.58^a^	0.11±0.13^a^	0.23±0.11^a^	0.12±0.06^a^	0.29±0.16^a^	3.02±2.62^a^
**ABCB1 3435C>T**									
**rs1045642**									
CC	26	440.4±210.7^a^	7.78±4.51^a^	0.98±0.36^a^	0.12±0.10^a^	0.26±0.23^a^	0.11±0.06^a^	0.23±0.14^a^	2.55±1.64^a^
CT	85	594.4±270.0^b^ *	8.56±3.88^a^	0.92±0.49^a^	0.10±0.06^a^	0.22±0.14^a^	0.12±0.10^a^	0.26±0.17^a^	2.98±2.57^a^
TT	34	525.4±253.3^a^	8.05±3.59^a^	1.00±0.66^a^	0.11±0.13^a^	0.21±0.15^a^	0.11±0.06^a^	0.24±0.16^a^	3.36±3.18^a^
**EPHX1 c.416A>G**									
**rs2234922**									
AA	80	542.8±251.4^a^	8.11±3.49^a^	1.00±0.62^a^	0.12±0.12^a^	0.24±0.19^a^ *	0.12±0.09^a^	0.26±0.17^a^ *	3.08±2.90^a^
AG	55	540.0±276.0^a^	8.16±4.27^a^	0.94±0.53^a^	0.09±0.05^a^	0.21±0.14^b^	0.11±0.08^a^	0.24±0.17^b^	3.95±2.44^a^
GG	10	510.0±202.5^a^	8.37±2.69^a^	1.05±0.29^a^	0.09±0.05^a^	0.13±0.08^b^	0.10±0.06^a^	0.13±0.08^b^	1.74±1.06^b^ *
**EPHX1 c.337T>C**									
**rs1051740**									
TT	63	523.2±257.3^a^	8.25±4.07^a^	0.94±0.49^a^	0.12±0.12^a^	0.26±0.16^a^	0.13±0.12^a^	0.31±0.22^a^ *	3.29±2.69^a^
TC	72	553.6±279.4^a^	8.07±3.96^a^	1.02±0.64^a^	0.10±0.07^a^	0.22±0.18^a^	0.11±0.09^a^	0.23±0.16^b^	2.86±2.74^a^
CC	10	620.0±147.6^a^	9.26±2.85^a^	0.76±0.15^b^ *	0.07±0.05^a^	0.18±0.17^a^	0.10±0.07^a^	0.25±0.17^b^	3.20±3.06^a^

^a,b^ Values sharing same letter are not significantly different.

*P<0.05

**P<0.01. (one way ANOVA analysis followed by Student’s T-test). CBZE: carbamazepine-10,11-epoxide; CBZD: 10,11-dihydroxy-carbazepine.

Whether these differences could be the consequence of a different pharmacodynamic response to CBZ in carriers with AA compared to GA or GG carriers, remains to be addressed. Furthermore, no significant difference in CBZ CL values associated with any of the SNPs investigated in this study, or with patients CBZ responsiveness/resistance, was observed (results not shown).

**Fig 4:**
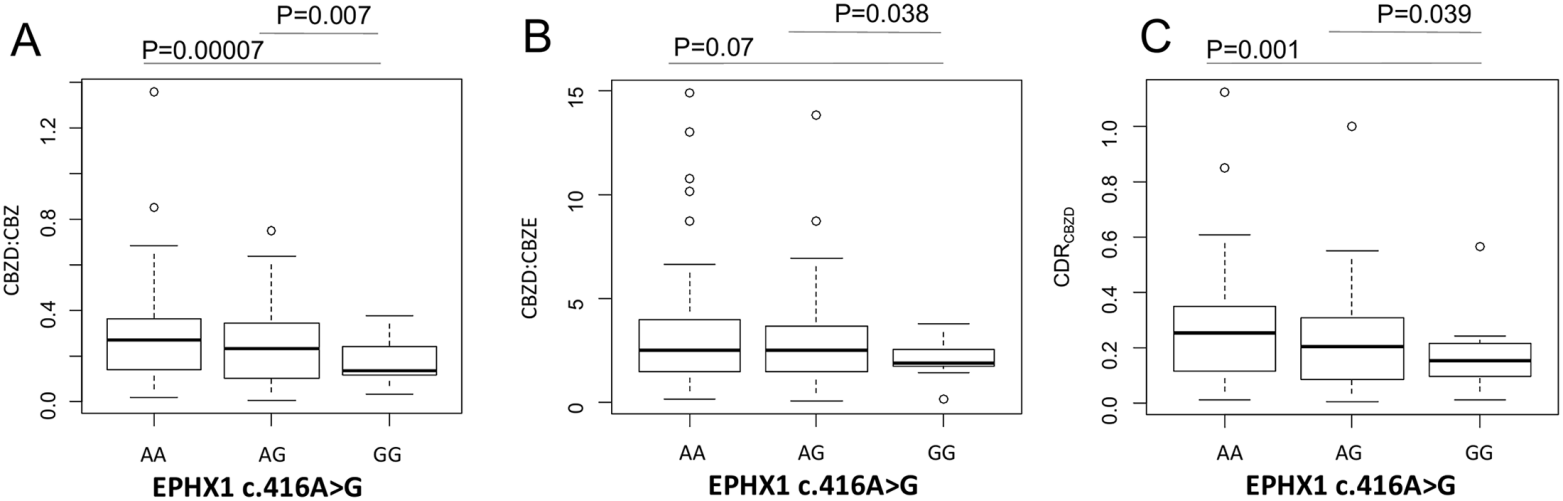
Graphical representations (box-plot) of the EPHX1 c.416A>G genotypes associations with (A) CBZD:CBZ, (B) CBZD:CBZE and (C) CDR_CBZD_,. CBZ: Carbamazepine; CBZD: Carbamazepine-10,11-trans dihydrodiol; CBZE: Carbamazepine-10,11-epoxide; CDR: Concentration/dose ratio. Data are mean ± standard deviations. Statistical significance for difference of means is shown (P values, one way ANOVA analysis followed by Student’s T-test).

**Fig 5:**
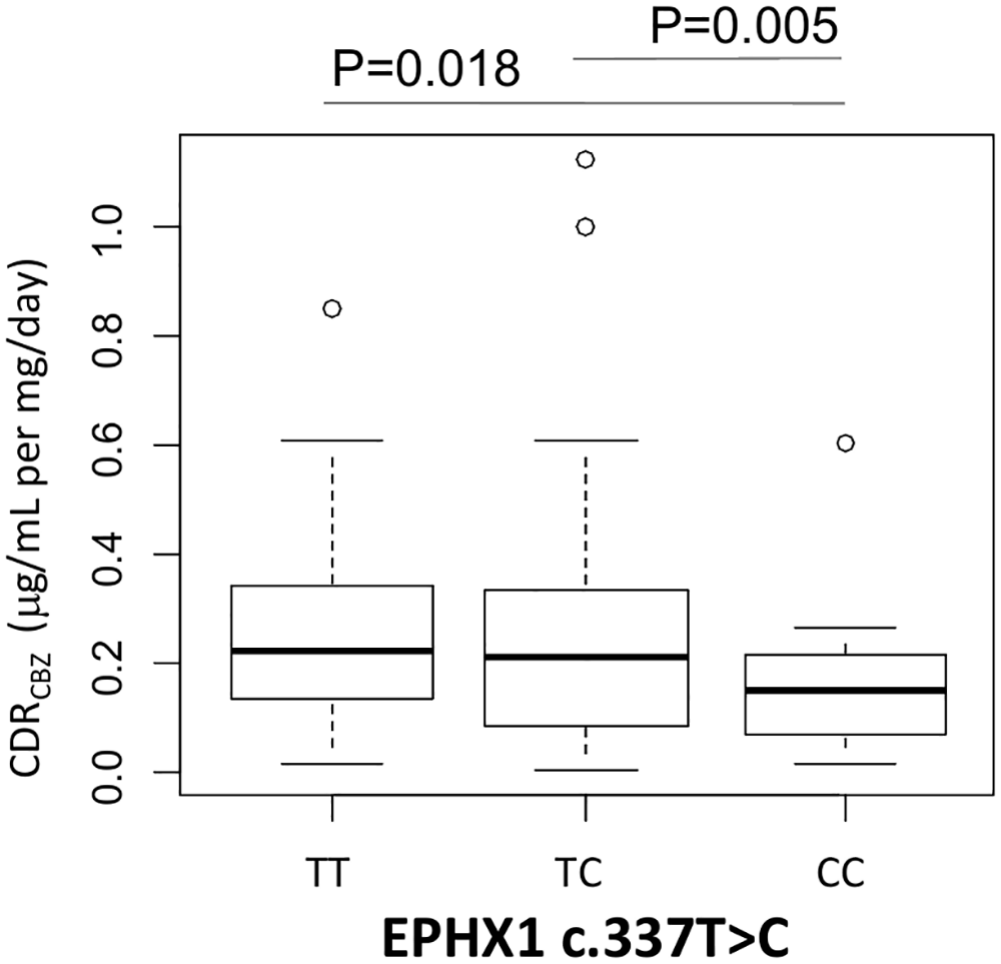
Graphical representations (box-plot) of the EPHX1 c.377T>C genotypes with CDR_CBZ_ CBZ: Carbamazepine; CDR: Concentration/dose ratio. Data are mean ± standard deviations. Statistical significance for difference of means is shown (P values, one way ANOVA analysis followed by Student’s T-test).

SCN1A c.3184A>G SNP does not affect CBZ metabolism (Table 2). The same observation applies to ABCB1 3435C>T, where differences in daily dosage of CBZ are lost following adjustment for body weight (Table 2). Similarly, plasma levels of CBZ and its metabolites were not affected by these two SNPs (Table 2). GG carriers of the EPHX1 c.416A>G SNP showed a reduced CBZ metabolism (mediated by CYP oxidation followed by microsomal epoxide hydrolase activity) compared to AA carriers as reflected by a significantly decreased ratio of CBZD to CBZ (0.13±0.08 to 0.26±0.17; P<0.05, Fig 4A), CBZD to CBZE ratio (1.74±1.06 to 3.08±2.90, P<0.05) (Fig 4B) and CDR_CBZD_ (0.13±0.08 to 0.24±0.19 16 μg/mL per mg/Kg; p<0.05, Fig 4C) (Table 2). It is worth noting that another SNP in EPHX1 (c.337T>C) affected CBZ plasma levels in carriers of the rare allele, showing significant lower CDR_CBZ_ compared to carriers of the wild type allele (TT 0.94±0.49 μg/mL per mg/Kg, CC 0.76±0.16 μg/mL per mg/Kg; P<0.05, Fig 5) (Table 2). In summary, SNPs in EPHX1 might affect microsomal epoxide hydrolase activity and plasma CBZ levels.

In addition, according to the report by Puranik et al. for a Caucasian population [17], haplotype analysis of EPHX1 gene was carried out and no significant association was found with the above parameters (S2 Fig).

### Assessment of CBZ resistance

**Fig 6:**
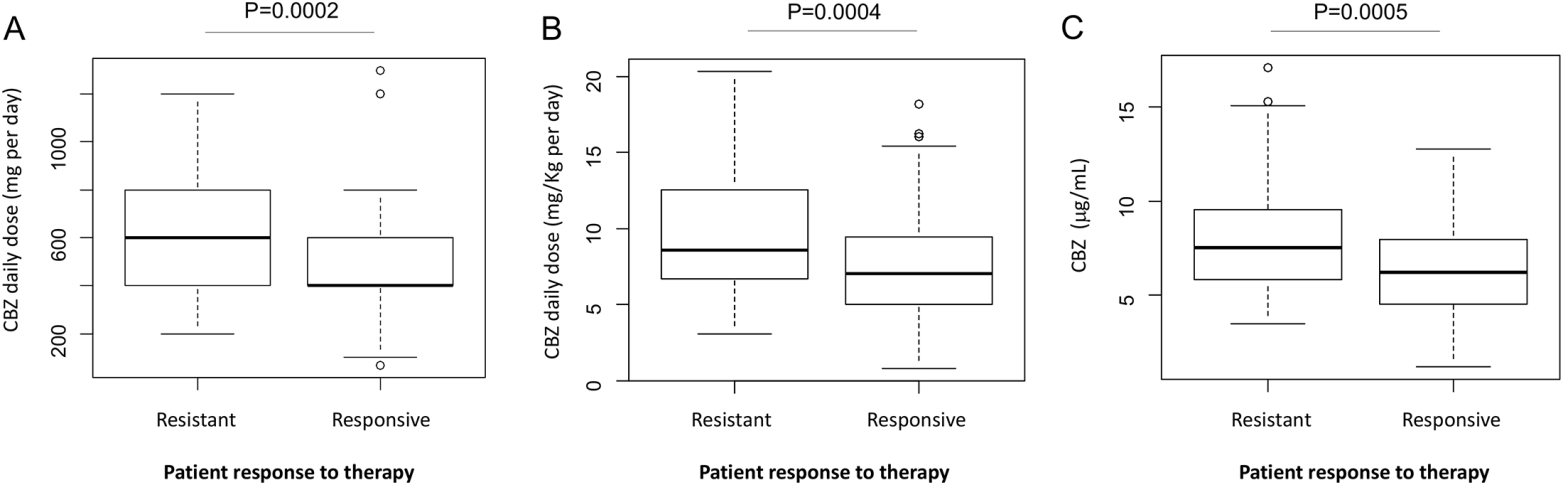
Graphical representations (box-plot) of the assessment of CBZ resistance due to patient response to therapy (A) CBZ daily dosage (mg/day), (B) CBZ maintenance dose (mg/kg per day) and (C) CBZ plasma concentration (μg/mL). Data are mean ± standard deviations. Statistical significance for difference of means is shown (P values, Student’s T-test).

Finally, we assessed whether the plasma levels of CBZ and its metabolites represent markers of responsiveness to CBZ treatment. While increased CBZ daily and maintenance doses in resistant patients compared to responsive patients (657±285 vs 489±231 mg/day; P<0.001) (Fig 6A and 6B) are expected, the observation that resistant patients also presented increased CBZ plasma levels (9.84±4.37 vs 7.41±3.43 μg/mL; P<0.001) (Fig 6C) is surprising.

To verify whether the differences in CBZ plasma levels were only due to the increased daily CBZ dose or maintenance dose, resistant and responsive patients were matched according to the dose of CBZ administered, by excluding n = 43 patients taking the lowest CBZ doses from the responsive group. The results of this analysis showed that CBZ plasma levels were still increased in resistant patients compared to responsive patients (S2 Table).

Indeed, none of the SNPs investigated here resulted in association with CBZ drug responsiveness in epilepsy patients (Table 3).

**Table 3:** Distribution of SCN1A, ABCB1, EPHX1 genes polymorphism in drug-resistant and drug-responsive epileptic patients.

SNPs	Gene/alleles	CBZ Resistance(n = 46)	CBZ Responsive (n = 99)	Odds Ratio (95% Cl)	P-Value
**SCN1A IVS5-91G>A**	**GG**	16 (34.8%)	34 (34.3%)	reference	
**rs3812718**	**GA**	25 (54.3%)	54 (54.6%)	0.98 (0.460, 2.104)	0.97
	**AA**	5 (10.9%)	11 (11.1%)	0.97 (0.287, 3.248)	0.96
	**GG**	16 (34.8%)	34 (34.3%)	reference	
	**GA+AA**	30 (65.2%)	65 (65.7%)	0.98 (0.470, 2.045)	0.97
**SCN1A c.3184A>G**	**AA**	8 (17.4%)	17 (17.2%)	reference	
**rs2298771**	**AG**	27 (58.7%)	56 (56.5%)	1.02 (0.393, 2.669)	0.96
	**GG**	11 (23.9%)	26 (26.3%)	0.90(0.300, 2.693)	0.85
	**AA**	8 (17.4%)	17 (17.2%)	reference	
	**AG+GG**	38 (82.6%)	82 (82.8%)	0.99 (0.391, 2.481)	0.97
**ABCB1 3435C>T**	**CC**	8 (17.4%)	18 (18.2%)	reference	
**rs1045642**	**CT**	28 (60.9%)	57 (57.6%)	1.11 (0.428, 2.851)	0.84
	**TT**	10 (21.7%)	24 (24.2%)	1.06(0.421, 2.643)	0.91
	**CC**	8 (17.4%)	18 (18.2%)	reference	
	**CT+TT**	38 (82.6%)	81 (81.8%)	1.06(0.421, 2.643)	0.91
**EPHX1 c.416A>G**	**AA**	25 (54.4%)	55 (55.6%)	reference	
**rs2234922**	**AG**	18 (39.1%)	37 (37.3%)	1.07(0.513, 2.233)	0.86
	**GG**	3 (6.5%)	7 (7.1%)	0.94(0.225, 3.950)	0.94
	**AA**	25 (54.4%)	55 (55.6%)	reference	
	**AG+GG**	21 (45.6%)	42 (43.8%)	1.05 (0.543,2.228)	0.79
**EPHX1 c.337T>C**	**TT**	23 (50%)	40 (40.4%)	reference	
**rs1051740**	**TC**	21 (45.6%)	51 (51.5%)	0.72 (0.348,1.474)	0.36
	**CC**	2 (4.4%)	8 (8.1%)	0.44(0.085,2.224)	0.31
	**TT**	23 (50%)	40 (40.4%)	reference	
	**TC+CC**	23(50%).	59 (59.6%)	0.68 (0.335, 1.370)	0.28

## Discussion

The present study investigated the effects of polymorphism of genes encoding the major drug transport, metabolizing enzymes and target proteins, on plasma concentrations of CBZ and its related metabolites (CBZE and CBZD). This was conducted in order to detect the interindividual variability in Kosovar patients of Albanian ethnicity with respect to CBZ pharmacodynamics, as well as its metabolism and disposition in patients with epilepsy.

Our results show that the mean concentrations of CBZ, CBZE and CBZD and their ratios in our cohort were comparable to those previously reported for other Oriental and Caucasian populations [17–19,21,28]. In addition, the correlation coefficients observed between daily dose and plasma CBZ and CBZE were in agreement with those reported by Krasniqi et al. for a German population of epileptic patients [33].

Our study has three key findings: (i) the SCN1A IVS5-91G>A SNP is associated with susceptibility to epilepsy, (ii) SNPs in EPHX1 gene influence CBZ pharmacokinetic and (iii) CBZ plasma level is not an indicator of resistance to the therapy.

The sodium channel α-subunit is the major binding site of several antiepileptic drugs. Therefore, the interest in genes encoding for this protein lies not only in the possible causal roles in epilepsy, but also in the potential effects on the antiepileptic drug efficacy.

There are several isoforms of α -subunits expressed in the brain, which are encoded by SCN1A, 2A, 3A and 8A [34,35]. Differential influence of genetic variants, namely SCN1A c.3184A>G and SCN1A IVS5-91, in epilepsy susceptibility and drug response have previously been reported [36–38]. These two SNPs were selected since, by belonging to a linkage disequilibrium block, they can be representative of other SNPs in the SCN1A gene [15,39].

Our results, which show increased mean CBZ maintenance dose, lower CDR_CBZ_ and higher CBZD:CBZ ratio in carriers of the IVS5-91G>AG variant of the SCN1A channel, are in agreement with previous findings in Caucasian patients [40,41]. Similar results were reported by Hung and colleagues, which showed in a Taiwanese population that carriers of the variant SCN1A IVS5–91G>A required higher CBZ dosages and lower ln(concentration–dose ratios) compared to noncarriers [15]. Recently, these patterns have been confirmed by Ma and colleagues in a population of Chinese patients [42]. Furthermore, increased doses of CBZ are associated with increased mean steady-state concentrations of CBZD [43], which could be linked to the effect of the SCN1A gene, IVS5–91 G>A variant, on CBZ dosage and CBZD:CBZ ratio.

Not all papers are in agreement with the findings above [44,45]. Whether ethnicity plays a role, remains to be addressed.

In addition, we also observed genetic variations in the genes encoding the expression of cerebral sodium channels in the epilepsy phenotypes compared to healthy control subjects, suggesting the involvement of this genotype in increased risk for developing epilepsy. These findings warrant further confirmation in future studies involving larger cohorts.

Previously, the impact of variants on a drug efflux transporter protein involved in the efflux of antiepileptic drugs, namely P-glycoprotein (Pgp; encoded by ABCB1 or MDR1), has been studied in different ethnic groups [46,47]. However, conflicting results have been reported, with some works indicating an impact on antiepileptic drug resistance [48–51], while others are in agreement with our observation, showing no effect of ABCB1 3435C>T on CBZ pharmacokinetic and drug response [52–55].

CYP3A4/A5 enzymes play a major role in the CBZ metabolism and in the onset of epilepsy pharmacoresistance [56–58]. Subsequent analyses aimed at addressing the contribution of CYP3A4 protein variants to the inter-individual variability of CYP3A4 activity were, however, less clear [59] or even observed a lack of effect of CYP3A4/5 variants on CBZ metabolism in both European Caucasian or in Asian populations [17,21,60,61].

For this reason, we decided to focus the investigation on the impact of SNPs in other genes that could explain CBZ pharmacoresistance beyond those in CYP3A4/A5 enzymes, including mEH, which has been proposed as a predictor of maintenance dose [27]

The human mEH, encoded by the EPHX1 gene, is expressed polymorphically [62]. The presence of two common variants, c.337T>C and c.416A>G, has been suggested to influence the catalytic activity of mEH in vitro and in vivo [63,64]. Further studies have shown that enzymatic expression levels and activity are altered and a significant association of EPHX1 SNPs with increased or decreased CBZD:CBZE ratios was found [15, 65–68], although this was not consistent in all studies [21]. While CBZD:CBZ is an indicator of enzymatic conversion of CBZ, dependent on both CYP enzymes and/or mEH, useful in determining unexpected CBZ levels [69], CBZD:CBZE ratio is considered as a sensitive indicator of mEH activity [15, 65–68]. This ratio was significantly lower in carriers of the variant EPHX1 c.416A>G, suggesting a reduced activity of the mEH, while the similarity of CBZE:CBZ ratio in carriers versus non carriers limits the relevance of differences in CYP enzyme activity.

We show here that variants in EPHX1 affect CBZ metabolism, either resulting in no effect on CDR_CBZ_ or reduced CBZD:CBZ, CBZD:CBZE ratios and CDR_CZBD_, which is in agreement with reports by Nakajima et al. [67] in the case of c.416A>G. We also observed that another EPHX1 variant (c.337T>C) is associated with lower CDR_CBZ_, in agreement with associations found by other authors [15,27]. However, as it is known that CBZ is not a direct substrate of mEH, the possibility of using CBZ plasma levels as a surrogate indicator for the evaluation of mEH activity has to be considered.

Previous studies have shown that SCN1A splice variants (encoding Na_v_ I.I channels) play a role in epilepsy susceptibility, with recent evidence of drug sensitivity due to tonic and use-dependent block of Na_v_1.1-5A and Na_v_1.1-5N, with therapeutically CBZ concentrations, showing more preferential activity for other reported AEDs than CBZ. [70,71]

Finally, we observed that resistant patients presented significantly increased daily dose, maintenance dose and plasma levels of CBZ. To exclude that the latter could simply be the consequence of increased administered dose, we compared responsive and resistant patients matched for the daily dose and still observed significantly increased plasma CBZ levels. Previous studies showed that in resistant patients access of CBZ to the brain was limited by the blood-brain barrier [72–74]. Whether this could, at least in part, explain our findings remains to be explored.

In conclusion, by showing a critical effect of polymorphisms in the response and efficacy of CBZ treatment in epileptic patients with a main focus on CBZ and metabolites, our work may set the stage for larger investigational studies aimed at evaluating the impact of pharmacogenomic approaches in the clinical management of patients with epilepsy.

## Supporting Information

S1 Dataset Original Study Dataset. (XLSX)

S1 Fig Correlation between CBZ daily dose and (a) plasma concentration of CBZ and (b) plasma correlation of CBZE. CBZE: carbamazepine-10,11-epoxide.
(TIF)

S2 Fig Graphical representation (boxplot) of the relationship between CBZD:CBZE ratio and EPHX1 SNPs (rs1051740-Tyr113His and rs2234922-His139Arg) diplotypes. P>0.05 (one way ANOVA). CBZ E: carbamazepine-10,11-epoxide; CBZD: 10,11-dihyroxy-carbamazepine.
(TIF)

S1 Table Distribution of SCN1A, ABCB1, EPHX1 genes polymorphisms; genotype and allele frequencies in epileptic patients vs. healthy subjects. (DOCX)

S2 Table CBZ daily dose, CBZ maintenance dose and CBZ plasma level stratified by response to CBZ therapy (responsive vs resistance patients) and corresponding P values for their difference of means (Student’s t-test). To match the average CBZ daily doses, n = 43 subjects with the lowest CBZ daily dosages were excluded from the analysis (P>0.05). Data are mean±standard deviation.
(DOCX)
