# Supplementary figures and images for: Polymorphic Variants of SCN1A and EPHX1 Influence Plasma Carbamazepine Concentration, Metabolism and Pharmacoresistance in a Population of Kosovar Albanian Epileptic Patients
Source: PLoS One. 2015 Nov 10;10(11):e0142408. doi: 10.1371/journal.pone.0142408 (PMC4640545; doi:10.1371/journal.pone.0142408)

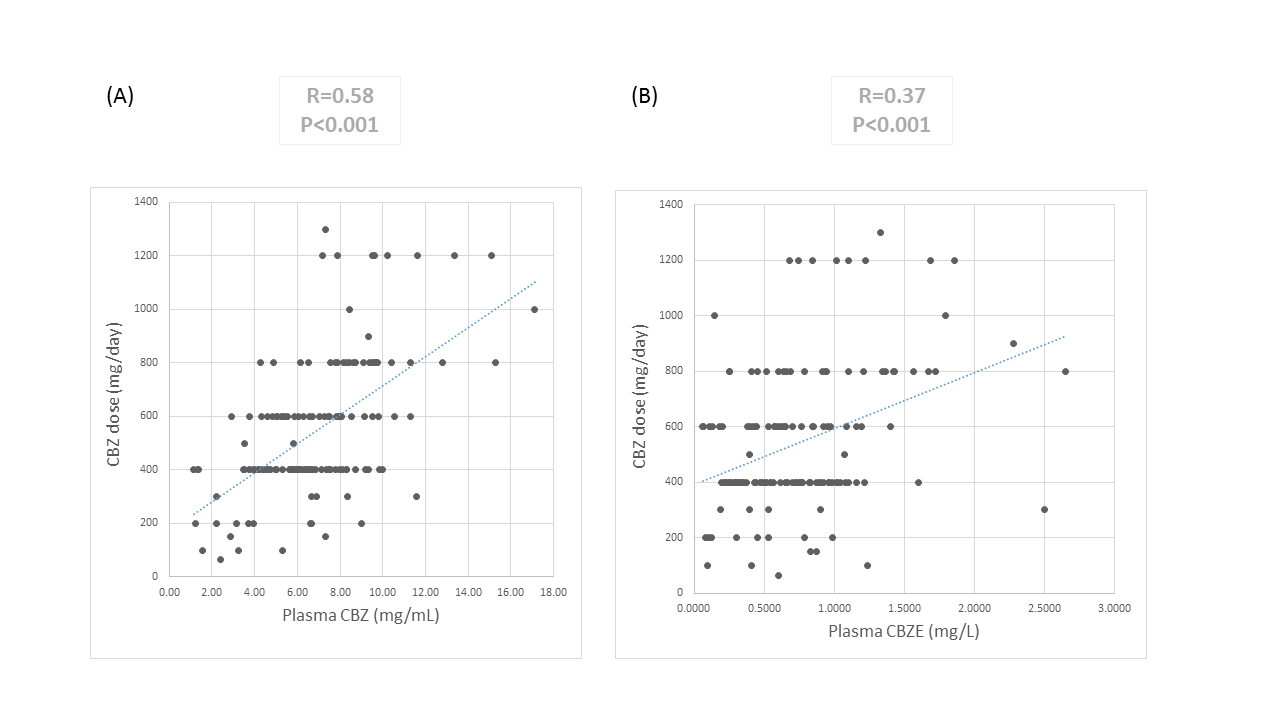

Supplement: S1 Fig — CBZE: carbamazepine-10,11-epoxide. (TIF) [file pone.0142408.s002.tif]

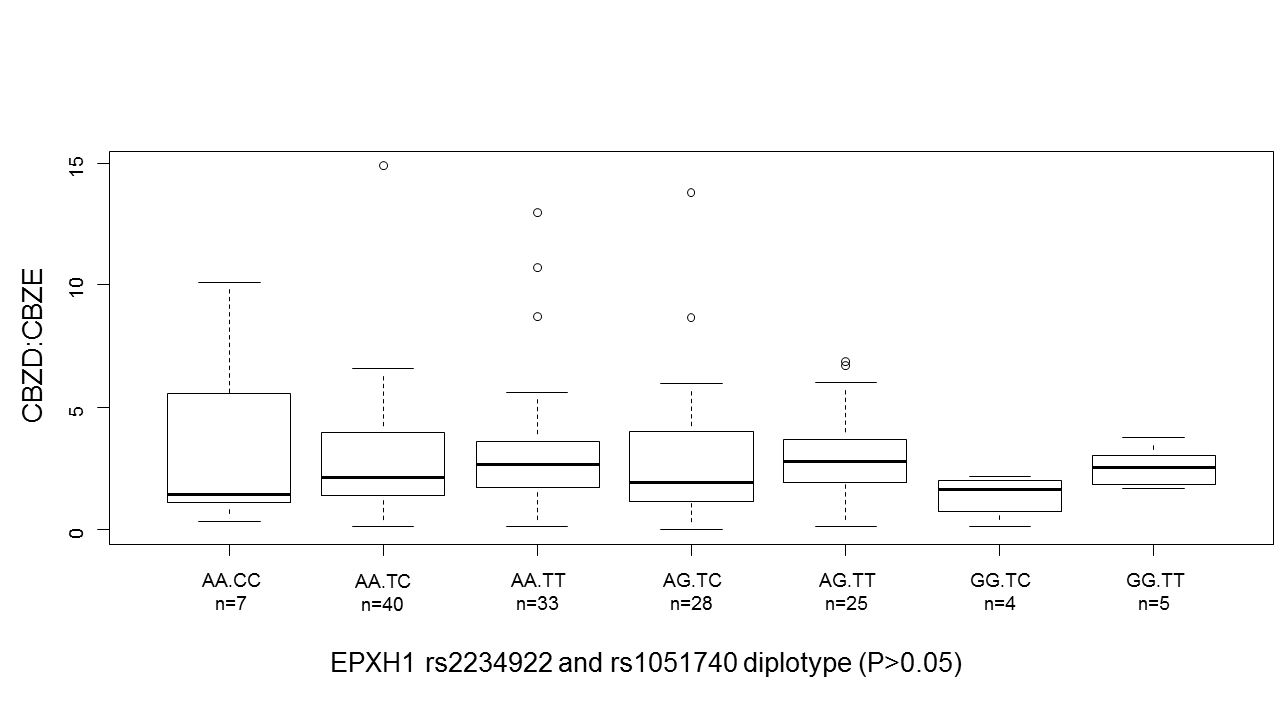

Supplement: S2 Fig — P>0.05 (one way ANOVA). CBZ E: carbamazepine-10,11-epoxide; CBZD: 10,11-dihyroxy-carbamazepine. (TIF) [file pone.0142408.s003.tif]
