# Supplementary material for: Polymorphic Variants of SCN1A and EPHX1 Influence Plasma Carbamazepine Concentration, Metabolism and Pharmacoresistance in a Population of Kosovar Albanian Epileptic Patients
Source: PLoS One. 2015 Nov 10;10(11):e0142408. doi: 10.1371/journal.pone.0142408 (PMC4640545; doi:10.1371/journal.pone.0142408)
Supplement: S1 Table — (DOCX) [file pone.0142408.s004.docx]

**S1 Table**. Distribution of SCN1A, ABCB1, EPHX1 genes polymorphisms; genotype and allele frequencies in epileptic patients vs. healthy subjects.

| **SNPs** | **Gene/alleles** | **Patients (n=145)** | **Controls (n=100)** | **Odds Ratio (95% Cl)** | **P-Value** |
| --- | --- | --- | --- | --- | --- |
| **SCN1A IVS5-91G>A** | **GG** | 50 (35%) | 49 (49%) | reference |  |
| **rs3812718** | **GA** | 79 (54%) | 43 (43%) | 1.80 (1.048, 3.094) | 0.033 |
|  | **AA** | 16 (11%) | 8 (8%) | 1.96 (0.769, 4.996) | 0.15 |
|  | **GG** | 50 (35%) | 49 (49%) | Reference |  |
|  | **AA+AG** | 95 (65%) | 51 (51%) | 1.83 (1.085, 3.072) | 0.023 |
| **SCN1A c.3184A>G** | **AA** | 25 (17.2%) | 16 (16%) | reference |  |
| **rs2298771** | **AG** | 83 (56.6%) | 54 (54%) | 0.98 (0.481, 2.011) | 0.96 |
|  | **GG** | 37 (26.2%) | 30 (30%) | 0.79 (0.358, 1.741) | 0.56 |
|  | **AA** | 25 (17%) | 16 (16%) | reference |  |
|  | **AG+GG** | 120 (83%) | 84 (84%) | 0.91 (0.460, 1.817) | 0.80 |
| **ABCB1 3435C>T** | **CC** | 26 (17.9%) | 29 (29%) | reference |  |
| **rs1045642** | **CT** | 85 (58.6%) | 39 (39%) | 2.43 (1.268,4.662) | 0.007 |
|  | **TT** | 34 (23.5%) | 32 (32%) | 1.19 (0.579,2.426) | 0.64 |
|  | **CC** | 26 (18%) | 29 (29%) | reference |  |
|  | **CT+TT** | 119 (82%) | 61 (61%) | 2.176 (1.179,4.0156) | 0.0129 |
| **EPHX1 c.416A>G** | **AA** | 80 (54.5%) | 58 (58%) | reference |  |
| **rs2234922** | **AG** | 55 (37.2) | 36 (36%) | 1.11 (0.646,1.899) | 0.71 |
|  | **GG** | 10 (8.3%) | 6 (6%) | 1.21 (0.416, 3.513) | 1.00 |
|  | **AA** | 80 (55%) | 58 (58%) | reference |  |
|  | **AG+GG** | 65 (45%) | 42 (42%) | 1.12 (0.671, 1.877) | 0.66 |
| **EPHX1 c.337T>C** | **TT** | 63 (42.8%) | 44 (44%) | reference |  |
| **rs1051740** | **TC** | 72 (50.3%) | 53 (53%) | 0.95 (0.5621, 1.601 ) | 0.84 |
|  | **CC** | 10 (6.9%) | 3 (3%) | 2.33 (0.606,8.949) | 0.22 |
|  | **TT** | 63 (43%) | 44 (44%) | reference |  |
|  | **TC+CC** | 82 (57%) | 56 (56%) | 1.23 (0.612,1.709 ) | 0.93 |
