# Supplementary material for: Polymorphic Variants of SCN1A and EPHX1 Influence Plasma Carbamazepine Concentration, Metabolism and Pharmacoresistance in a Population of Kosovar Albanian Epileptic Patients
Source: PLoS One. 2015 Nov 10;10(11):e0142408. doi: 10.1371/journal.pone.0142408 (PMC4640545; doi:10.1371/journal.pone.0142408)
Supplement: S2 Table — To match the average CBZ daily doses, n = 43 subjects with the lowest CBZ daily dosages were excluded from the analysis (P>0.05). Data are mean±standard deviation. (DOCX) [file pone.0142408.s005.docx]

**S2 Table.** CBZ daily dose, CBZ maintenance dose and CBZ plasma level stratified by response to CBZ therapy (responsive vs resistance patients) and corresponding P values for their difference of means (Student’s t-test). To match the average CBZ daily doses, n=43 subjects with the lowest CBZ daily dosages were excluded from the analysis (P>0.05). Data are mean±standard deviation.

|  | **Daily dose** | **maintenance Dose** | **Plasma CBZ** |
| --- | --- | --- | --- |
| **Responsive (n=56)** | 619.6 (211.8) | 8.80 (2.91) | 6.79 (2.39) |
| **Resistant (n=46)** | 656.5 (284.9) | 9.84 (4.37) | 7.94 (3.02) |
| P= | 0.234 | 0.086 | 0.019 |
